# Network study of responses to unusualness and psychological stress during the COVID-19 outbreak in Korea

**DOI:** 10.1371/journal.pone.0246894

**Published:** 2021-02-26

**Authors:** Seunghyong Ryu, In-Hoo Park, Mina Kim, Yu-Ri Lee, Jonghun Lee, Honey Kim, Min Jhon, Ju-Wan Kim, Ju-Yeon Lee, Jae-Min Kim, Sung-Wan Kim

**Affiliations:** 1 Department of Psychiatry, Chonnam National University Medical School, Gwangju, Korea; 2 Gwangju Mental Health and Welfare Commission, Gwangju, Korea; 3 Department of Social Welfare, Nambu University, Gwangju, Korea; 4 Daegu Metropolitan Mental Health and Welfare Center, Daegu, Korea; 5 Department of Psychiatry, Catholic University of Daegu, College of Medicine, Daegu, Korea; UCLA Fielding School of Public Health, UNITED STATES

## Abstract

The dramatic changes in people’s daily lives caused by the 2019 coronavirus (COVID-19) pandemic have had a huge impact on their emotions and behaviors. This study aimed to examine psychosocial responses to COVID-19 using network analysis. A total of 1,500 urban residents of South Korea, selected from an online public panel, were surveyed using self-rating questionnaires addressing daily life changes, fear of infection, and distress related to COVID-19. Participants also completed a 10-item Perceived Stress Scale survey. We constructed regularized partial correlation networks, estimated global and local network metrics, tested network accuracy and stability, and compared the estimated networks between men and women. The network of the psychosocial responses consisted of 24 nodes that were classified into five groups: ‘fear of infection’, ‘difficulty with outside activities’, ‘economic loss’, ‘altered eating and sleeping’, and ‘adaptive stress’. The node centralities indicated that ‘distress in obtaining daily necessities’ and ‘concern about harming others’ were the most important issues in people’s responses to COVID-19. These nodes were connected by a negative edge, reflecting individual- and community-level issues, respectively. The overall level of perceived stress was linked to the network by the connection node ‘anger toward others or society’, which was associated with economic problems in men, but with distress from changes in daily activities in women. The results suggest that two contrasting feelings—personal insecurity regarding basic needs and a collectivistic orientation—play roles in the response to unusual experiences and distress due to COVID-19. This study also showed that public anger could arise from the psychological stress under the conditions imposed by COVID-19.

## 1. Introduction

The novel coronavirus pandemic, officially designated the coronavirus disease 2019 (COVID-19) by the World Health Organization, has been a major global health crisis. In South Korea, the first patient with COVID-19, who flew in from Wuhan, China, was identified on January 20, 2020. Soon thereafter, the number of confirmed patients began to increase rapidly, mainly in Daegu City and nearby areas, after identification of community spread originating from a religious cult at the end of February [1,2]. In early April, the number of confirmed cases had exceeded 10,000 nationwide in South Korea. To date, as of the writing of this work, the case count continues to increase, albeit more gradually.

In the absence of effective vaccines and treatments, the high infectivity and mortality of COVID-19 have introduced huge changes to people’s daily lives [3]. The dramatic increase in the number of patients has resulted in severe shortages of medical resources, personnel, and supplies [4]. Accordingly, government authorities have had to implement stringent public health measures to curtail the spread of COVID-19, including quarantining those in close contact with a patient or overseas entrant, restricting public gatherings, shutting down schools, and requiring mandatory wearing of face masks in public facilities, as well as other social distancing policies. Some have felt that these measures have violated individual freedoms and rights, despite their being intended for the collective good [5]. In addition, people are fearful of contracting COVID-19. In an effort to protect themselves and their families, they often wait in long lines, while social distancing, to buy face masks. Most have stayed at home unless absolutely necessary (e.g., for work, medical treatment, or supplies). The continuation of this situation has triggered a global economic downturn, leading to poor company performance, increased unemployment, and reduced incomes for many [6]. The extent of the damage to society from COVID-19 has never been seen before. The emotional stress and maladaptive behaviors of the general population have further revealed and exacerbated existing social and mental health problems [7–9], while introducing many more.

In the wake of the COVID-19 outbreak, people continue to experience stress in adapting to the extreme circumstances under pandemic conditions. Some studies have investigated the impact of COVID-19 on the mental health in the general population and in healthcare workers [10–12]. However, the results have been limited to describing people’s emotions, thoughts, and behaviors, separately, with respect to the pandemic. Given that behaviors are determined by complex interactions between various factors of an individual and others, we attempted to examine people’s experiences during the COVID-19 outbreak in a more integrated way.

Network analysis is a powerful methodological approach that can be used to estimate complex patterns of interconnected variables and obtain visual depictions of system-level relationships [13]. Thus, we aimed to explore the most influential issues affecting people’s behavioral and psychological changes in response to COVID-19 and delineate how the unusual experiences associated with the pandemic have added to people’s psychological burden using network models. To this end, in the present study, we performed network analyses to explore the interactions between daily life changes, fear of infection, and distress during the COVID-19 outbreak in South Korea with the aim of examining how unusual experiences and stress responses have changed people’s lives. Then, we investigated the relationship between psychosocial responses to COVID-19 and the study participants’ levels of psychological stress from a network perspective. In addition, considering evidence of sex differences in the levels of perceived stress [14,15], we compared network model results between men and women.

## 2. Materials and methods

### 2.1. Participants

Between April 24 and May 5, 2020, the third month of the COVID-19 outbreak in South Korea, we conducted an online survey of the general population with participant ages ranging from 19 to 65 years. Three geographic areas were surveyed: the Seoul metropolitan area (Seoul Capital and Gyeonggi Province around Seoul, N = 500), Daegu City (N = 500), and Gwangju City (N = 500). Participants were recruited using a quota sampling method with identical distributions of age and gender among these regions. All participants were selected from panels of an online survey service provider (Macromill Embrain), which has 1,324,315 people available to take part in surveys, all of whom have individual identification numbers. Participants can only take part after providing consent for the use of personal information. For this survey, an email invitation was sent to 4,065 people in the study regions, and participation was voluntary. The informed consent form could be accessed by clicking on a link on the survey page. Following the provision of consent, participants indicated their age and area of residence; if they met the selection criteria, the survey began. Survey responses could not be reviewed or changed using the browser’s back button. When a participant clicked the “submit” button on the final page, the survey was considered complete and could not be repeated. A total of 1,819 people completed the questionnaire; 319 were excluded from the final analysis because they responded with the same answer option throughout, responded too quickly, or for other reasons. We ultimately obtained data for 1,500 respondents.

This research was reviewed and approved by the Institutional Review Board of Chonnam National University Hospital (CNUH-2020-092). Electronic informed consent was obtained from each participant prior to starting the investigation.

### 2.2. Assessments

We designed three self-rating questionnaires on daily life changes, fear of infection, and distress related to the COVID-19 outbreak (Table 1). The items were based on the existing literature and our experience. All items were rated using a Likert scale from 1 (strongly disagree) to 5 (strongly agree). First, participants were asked about changes in their daily life after the COVID-19 outbreak, which included nine items on questionnaire A: ‘reluctance to use public transportation (A1),’ ‘trouble going out (A2),’ ‘lower income (A3),’ ‘setbacks with personal schedule (A4),’ ‘setbacks with official schedule (A5),’ ‘irregular diet (A6),’ ‘eating instant food more often (A7),’ ‘irregular sleep schedule (A8),’ and ‘less exercise time (A9).’ Next, they were asked about their concerns about COVID-19 in nine items on questionnaire B: ‘concern about being infected (B1),’ ‘concern about family being infected (B2),’ ‘fear of dying (B3),’ ‘concern about asymptomatic infected people (B4),’ ‘concern about being quarantined (B5),’ ‘concern about disclosure of movement (B6),’ ‘concern about being blamed by others (B7),’ ‘concern about harming others (B8),’ and ‘concern about deteriorating health due to restrictions on hospital visits (B9).’ Finally, they were asked to consider their distress related to COVID-19 across six items on questionnaire C: ‘distress obtaining masks or hand sanitizers (C1),’ ‘distress obtaining daily necessities (C2),’ ‘economic stress (C3),’ ‘distress from family support (C4),’ ‘distress from changes in daily activities (C5),’ and ‘anger toward others or society (C6).’ In addition, the participants completed the Korean version of the 10-item Perceived Stress Scale (PSS-10) to assess their perceptions of psychological stress during the COVID-19 outbreak [16,17]. This scale measured the degree to which life over the past several months during the COVID-19 pandemic had been experienced as being unpredictable, uncontrollable, and overwhelming on a 5-point Likert scale. After reversing the scores of the four positively stated items, a PSS-10 total score was obtained by summing up all 10 items. A high total score indicates a high level of perceived psychological stress.

**Table 1:** Daily life changes, fear, and distress related to the COVID-19 outbreak in 1,500 Koreans.

Survey items on questionnaires*		Score**	Cronbach’s alpha
Questionnaire A for daily life changes after the COVID-19 outbreak			
A1	I am reluctant to use public transportation to and from school and commuting.	4 (4–5)	0.761
A2	Because of the fear of infection, I am limited to where I can go when I go out (eating out, movies, shopping, and so forth).	5 (4–5)	
A3	In the aftermath of COVID-19, I have experienced a loss in income.	3 (2–4)	
A4	COVID-19 is disrupting my personal itinerary and planning (travel, vacation, and so forth).	5 (4–5)	
A5	COVID-19 is disrupting official schedules and plans (business trips, workshops, exams, classes, and so forth).	4 (4–5)	
A6	My diet has become irregular since the COVID-19 outbreak.	2 (2–3)	
A7	I eat instant food more often since the COVID-19 outbreak.	3 (2–4)	
A8	My sleep schedule has become irregular since the COVID-19 outbreak.	3 (2–4)	
A9	The amount of time I spend exercising has decreased since the COVID-19 outbreak.	4 (3–5)	
Questionnaire B for fears/concerns about COVID-19			
B1	I am afraid I will get COVID-19.	4 (3–5)	0.881
B2	I am afraid my family will get COVID-19.	4 (4–5)	
B3	I am afraid my family or I will die from COVID-19.	4 (3–4)	
B4	I am afraid there will be asymptomatic infected people around me.	4 (3–4)	
B5	I am afraid I will be quarantined for two weeks on contact with an infected person.	4 (3–5)	
B6	I am afraid all of my movements (places/people visited) will be revealed when I am confirmed.	4 (2–4)	
B7	I am afraid I will be blamed by others when I am infected.	4 (3–5)	
B8	I am afraid when I am infected that I will infect others around me.	5 (4–5)	
B9	I am afraid my health will deteriorate due to restrictions on hospital visits.	3 (3–4)	
Questionnaire C for distress related to the COVID-19 outbreak			
C1	I experience distress from difficulties in obtaining masks or hand sanitizers	3 (2–4)	0.788
C2	I experience distress from difficulties in obtaining daily necessities such as food or toilet paper	2 (1–3)	
C3	I am experiencing economic stress (increased economic burden due to less income or more inflation)	4 (3–4)	
C4	I am distressed due to family responsibilities (increased family care or parenting burden)	3 (2–4)	
C5	I am distressed due to changes in daily activities (canceling appointments with friends, reducing external activities, and so forth)	4 (3–4)	
C6	I experience feelings of anger/resentment toward others or society	3 (2–4)	

*All items on the questionnaires were rated using a 5-point Likert scale (1, strongly disagree; 2, disagree; 3, neutral; 4, agree; 5, strongly agree).

**Median (interquartile range).

### 2.3. Network construction

All analyses were conducted using R (version 3.6.1, https://www.r-project.org/). First, we assumed a network of responses to unusualness consisting of the 24 items from the three questionnaires (A, B, and C) on daily life changes, fear, and distress related to COVID-19 represented as nodes A1–A9, B1–B9, and C1–C6, respectively, and the associations between nodes represented as edges. For the edges, we calculated polychoric correlations for each pair of nodes using ‘cor_auto,’ a function of the R package ‘qgraph’ [18], which computes a correlation matrix based on polychoric, polyserial, and Pearson correlations. Then, using the polychoric correlations as input, we estimated a regularized partial correlation network via the Extended Bayesian Information Criterion (EBIC) graphical Least Absolute Shrinkage and Selection Operator (LASSO), which shrinks edges via regularization and sets very small edges to zero, producing a sparse and parsimonious network model [19,20]. We carried out this process using the R package ‘qgraph’ [18], which automatically implements graphical LASSO regularization in combination with EBIC model selection. At that time, we set the value of the hyperparameter γ, which denotes the strength of the EBIC’s preference for sparser models, to 0.5. This reflects a balance between a spurious network (γ = 0) and a more parsimonious network (γ = 1) [21]. The nodes were positioned using the Fruchterman–Reingold algorithm [22]. This algorithm calculates the optimal layout for placing nodes with strong connections close to each other, while placing those with weaker and fewer connections farther apart.

Then, we investigated statistical communities among the 24 nodes using the exploratory graph analysis (EGA) in the R package ‘EGAnet’ [23], which estimates the number of dimensions or factors using graphical LASSO or a triangulated maximally filtered graph and community detection algorithms. We applied the Walktrap algorithm, a node similarity-based approach, to the EGA as a community detection algorithm [24].

Finally, we added the PSS-10 total score as an extra node (PSS) to construct an extended network integrating responses to unusualness and overall level of psychological stress experienced by participants during the COVID-19 outbreak. To do this, we calculated polyserial correlations for the PSS node using the same method as described above and drew its structure as a flow diagram showing how the PSS node was connected to other nodes of responses to unusualness due to COVID-19 using the ‘flow’ function of the R package ‘qgraph’ [18].

### 2.4. Global and local network metrics

We calculated several macroscopic properties and the overall connectedness of the network using the R package ‘igraph,’ as follows [25]. The network density is the actual number of edges as a proportion of the total possible number of edges [26]. The average shortest path length, the mean of the shortest path lengths between all pairs of nodes in the network, is also an important measure of a network’s overall connectedness [27]. The average clustering coefficient, i.e., the mean of the local clustering coefficients, which measure the probability that any two neighbors of a node are connected, indicates the extent to which nodes tend to cluster together [28]. For example, a network with a higher density and average clustering coefficient, as well as a lower average shortest path length, can be considered more tightly interconnected [29].

Centrality indices provide insight into the relative importance of a node in the context of the other nodes in the network [30,31]. In general, central nodes with high centrality indices in a network are the most influential and can propagate their information content easier and faster than other nodes [32,33]. Using the R package ‘qgraph’ [18], we assessed three centrality indices for each node within the network: strength, closeness, and betweenness. Strength centrality is calculated by taking the sum of all absolute edge weights a node is directly connected to, indicating the strength of a node’s direct connections to other nodes [34]. Closeness centrality is the inverse of the average of the shortest path length from a node to all other nodes in the network; it indicates how close the node is to all other nodes in the network [30]. In other words, a node with high closeness centrality is more likely to quickly affect other nodes, and changes in other nodes are more likely to affect nodes with high closeness centrality [35]. Betweenness centrality measures the number of times that a node lies on the shortest path between two other nodes [36]. A node with high betweenness centrality can influence the information flow between non-directly connected nodes and has an important intermediary position [35]. In addition, we tested for significant differences between nodes in centrality indices using bootstrapped difference tests in the R package ‘bootnet’ [37].

### 2.5. Network accuracy and stability

Following the method proposed by Epskamp *et al*. [37], we examined the accuracy and stability of the network using the R package ‘bootnet.’ First, to assess the robustness of the edge weights, we drew 2,500 bootstrapped confidence intervals (CIs) of the edge weights. The bootstrapped CIs of edge weights should not be interpreted as the significance of an edge being different from zero, but only used to show the accuracy of edge weight estimates and to compare edges to one another [37]. When a bootstrapped CI around a specific edge weight does not overlap with the CIs of other edge weights, this indicates that the weight of this edge differs significantly from other edge weights. Thus, larger bootstrapped CIs indicate lower stability in the estimation of the edge weights, which implies that the strength of edges should be interpreted with caution. Then we investigated the stability of the centrality indices after observing only portions of the data using a case-dropping subset bootstrap (2,500 iterations), which determines how many samples can be removed from the network before the results become unstable [37,38]. This measure is quantified by the correlation stability (CS) coefficient. The CS coefficient represents the maximum proportion of cases that can be dropped while retaining a correlation of 0.7 between the original centrality indices and centrality based on a subset [37]. It has been suggested that the CS coefficient should be at least 0.25, preferably above 0.5 [38].

### 2.6. Network comparison

In addition, we constructed the network structures of men and women and compared them using the R package ‘NetworkComparisonTest’ [39], a permutation-based hypothesis test in which the difference between the networks of two groups is calculated repeatedly for randomly regrouped individuals. We performed 2,500 iterations to assess the difference between the two networks through comparisons of network structure, global strength, and centrality and edge invariance.

## 3. Results

### 3.1. Demographic characteristics and responses to the questionnaires

Table 1 presents the scores for individual items on the three questionnaires for daily life changes, fear, and distress related to COVID-19, as well as the internal consistency of items among each questionnaire. Participants showed a strong agreement on the ‘trouble going out’ (A2 item), ‘setbacks with personal schedule’ (A4 item), and ‘concern about harming others’ (B8 item) with a median score of 5 (strongly agree), while they generally disagreed with ‘irregular diet’ (A6 item) and ‘distress in obtaining daily necessities’ (C2 item), with a median score of 2 (disagree). Each questionnaire showed high internal consistency (Cronbach alpha = 0.761, 0.881, and 0.788 for questionnaires for daily life changes, fear, and distress, respectively).

Table 2 summarizes the demographic characteristics of the 1,500 participants, in which 50% of the participants were men, with an average age of 40.2 ± 11.9 years. Of the 1,500 participants, 49.3% were married, and 69.3% had a job.

**Table 2:** Demographic characteristics of the study population.

Variable	Value*
Sex, Men	750 (50.0)
Age, years	40.2 ± 11.9 (19 ‒ 65)
19‒29	360 (24.0)
30‒39	360 (24.0)
40‒49	360 (24.0)
50‒65	420 (28.0)
Education	
High school or lower	256 (17.1)
College or university	1094 (72.9)
Graduate school	150 (10.0)
Marital status	
Single	717 (47.8)
Married	740 (49.3)
Divorced	36 (2.4)
Bereaved/widowed	7 (0.5)
Employment status	
Employed	1039 (69.3)
Unemployed	158 (10.5)
Stay-at-home	169 (11.3)
Student	134 (8.9)
Medical insurance	
National health insurance	1431 (95.4)
Medicare	69 (4.6)

*Data are presented as the mean ± the standard deviation (range) or number (%).

### 3.2. Network of the psychosocial response to COVID-19

**Fig 1:**
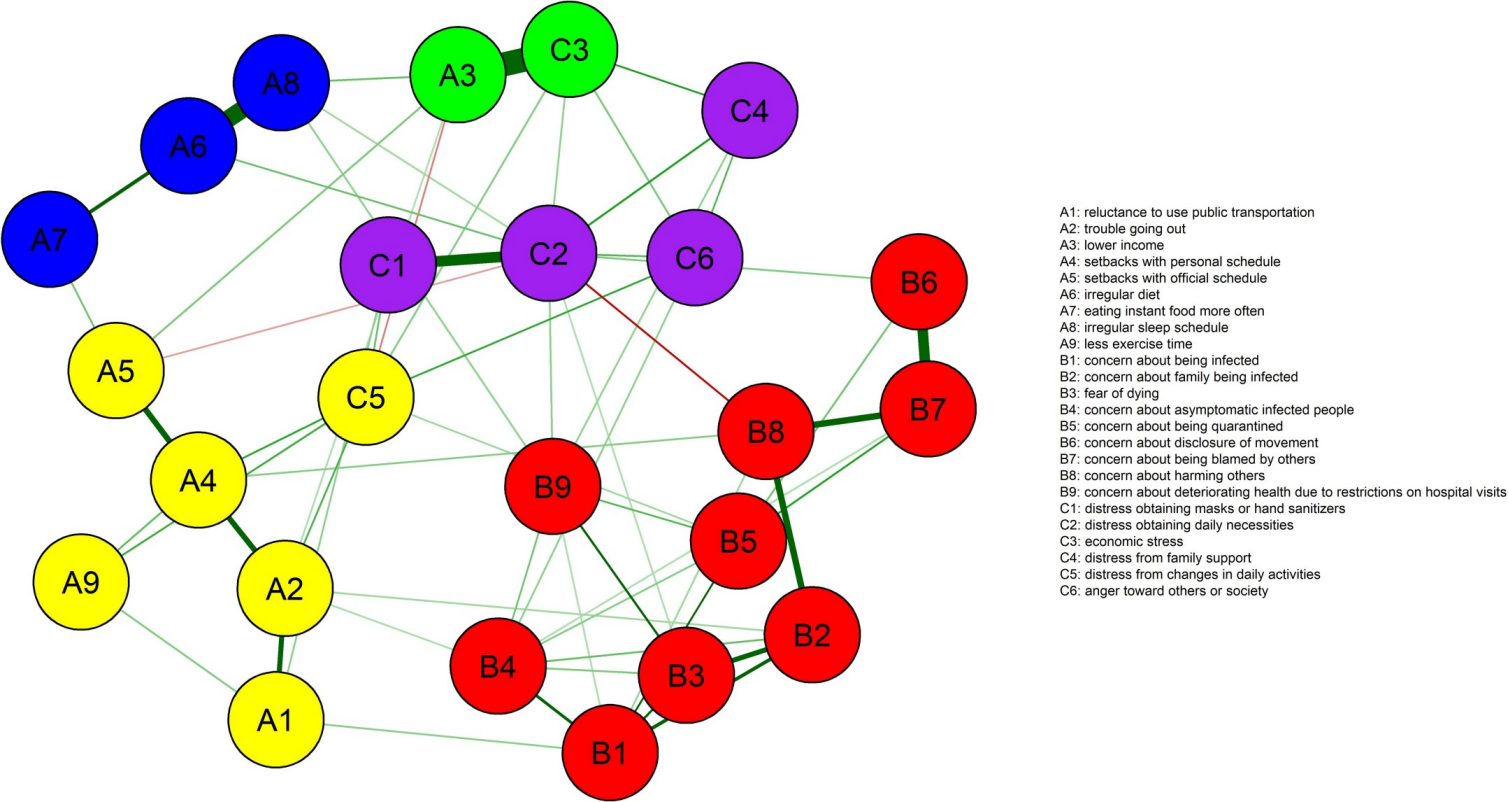
Network of the responses to unusualness due to COVID-19 based on a regularized partial correlation via the extended Bayesian information criterion graphical least absolute shrinkage and selection operator. The 24 nodes are grouped into five communities of ‘fear of infection’ (in red), ‘difficulty in outside activities’ (yellow), ‘economic loss’ (green), ‘disturbance in eating and sleeping’ (blue), and ‘adaptive stress’ (purple). The thickness of the edges represents the magnitude of the correlation. Positive correlations are displayed as green and negative correlations are displayed as red.

The resulting 24-node network of the responses to unusualness during the COVID-19 outbreak is presented in Fig 1. The network was well connected, with no isolated nodes. The EGA grouped the 24 nodes into five communities: ‘fear of infection’ (B1 to B9), ‘difficulty in outside activities’ (A1, A2, A4, A5, A9, and C5), ‘economic loss’ (A3 and C3), ‘disturbance in eating and sleeping’ (A6, A7, and A8), and ‘adaptive stress’ (C1, C2, C4, and C6). The network had a network density of 0.225, an average shortest path length of 2.036, and an average clustering coefficient of 0.300.

**Fig 2:**
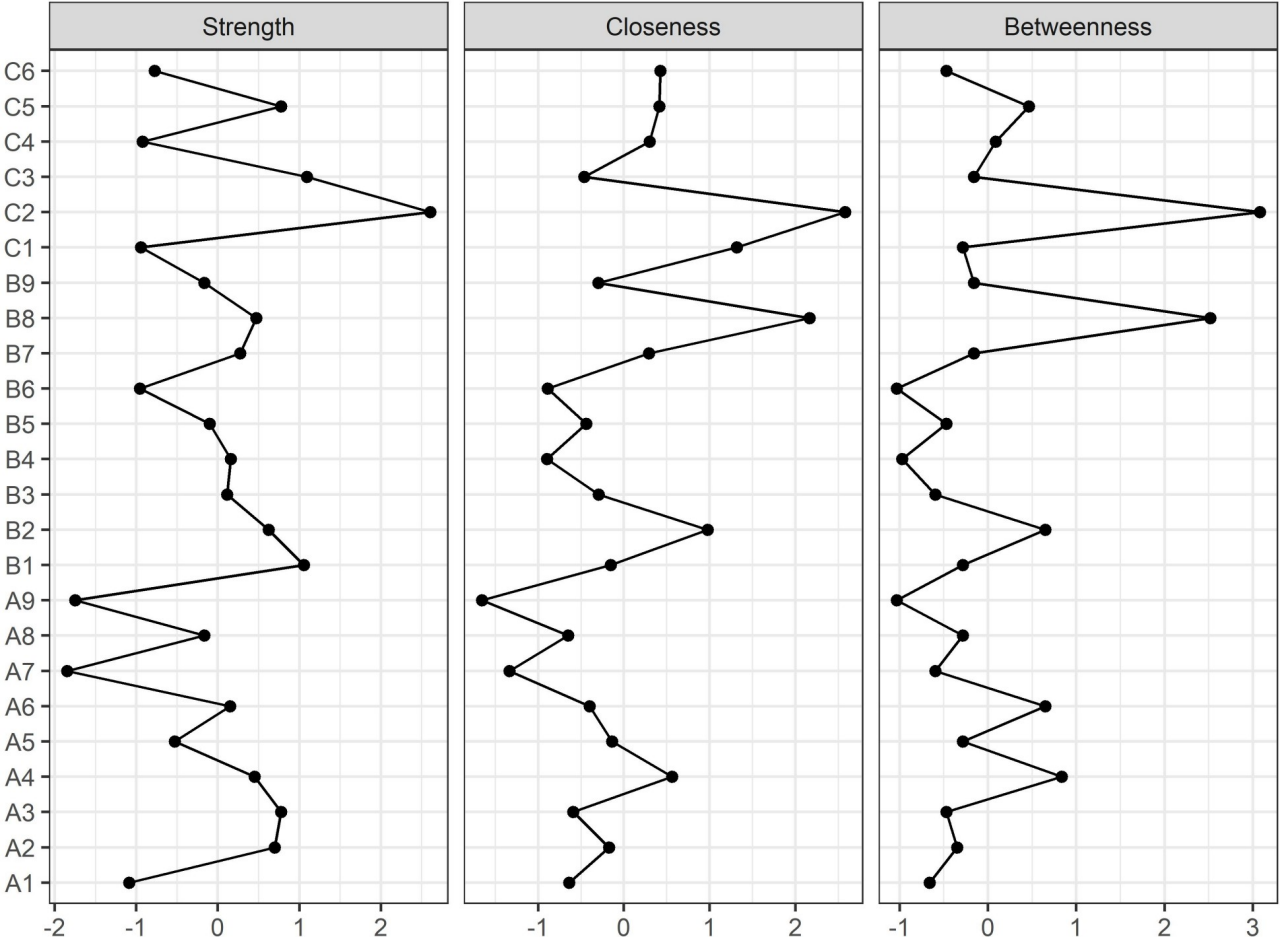
Centrality indices of the network of responses to unusualness due to COVID-19. Centrality indices are shown as standardized z-scores.

Fig 2 depicts the standardized centrality indices of the network. In terms of node strength, closeness, and betweenness, the ‘distress in obtaining daily necessities’ (C2 node) was the most central domain of the responses to unusualness related to COVID-19. The ‘concerns about harming others’ (B8 node) also showed high levels of closeness and betweenness centrality, comparable to those of the C2 node. The bootstrap difference test revealed significant differences between the C2 node and most other nodes for all centrality indices (S1 Fig). Node B8 was also significantly different from most other nodes in terms of closeness and betweenness centrality.

In examining edge weight accuracy, the bootstrapped CIs around the edge weights (estimated to be larger than zero) were narrow overall, indicating a fair degree of accuracy in the estimation of edge weights (S2 Fig). With regard to the stability of the centrality indices (S3 Fig), the results of the case-dropping subset bootstrap revealed a correlation stability (CS) coefficient for a node strength of 0.595, which exceeded the proposed threshold of 0.5. In addition, the CS coefficients for node closeness and betweenness were 0.283 and 0.361, respectively, slightly above the minimum acceptable level.

### 3.3. Relationship between responses to unusualness and psychological stress related to COVID-19

**Fig 3:**
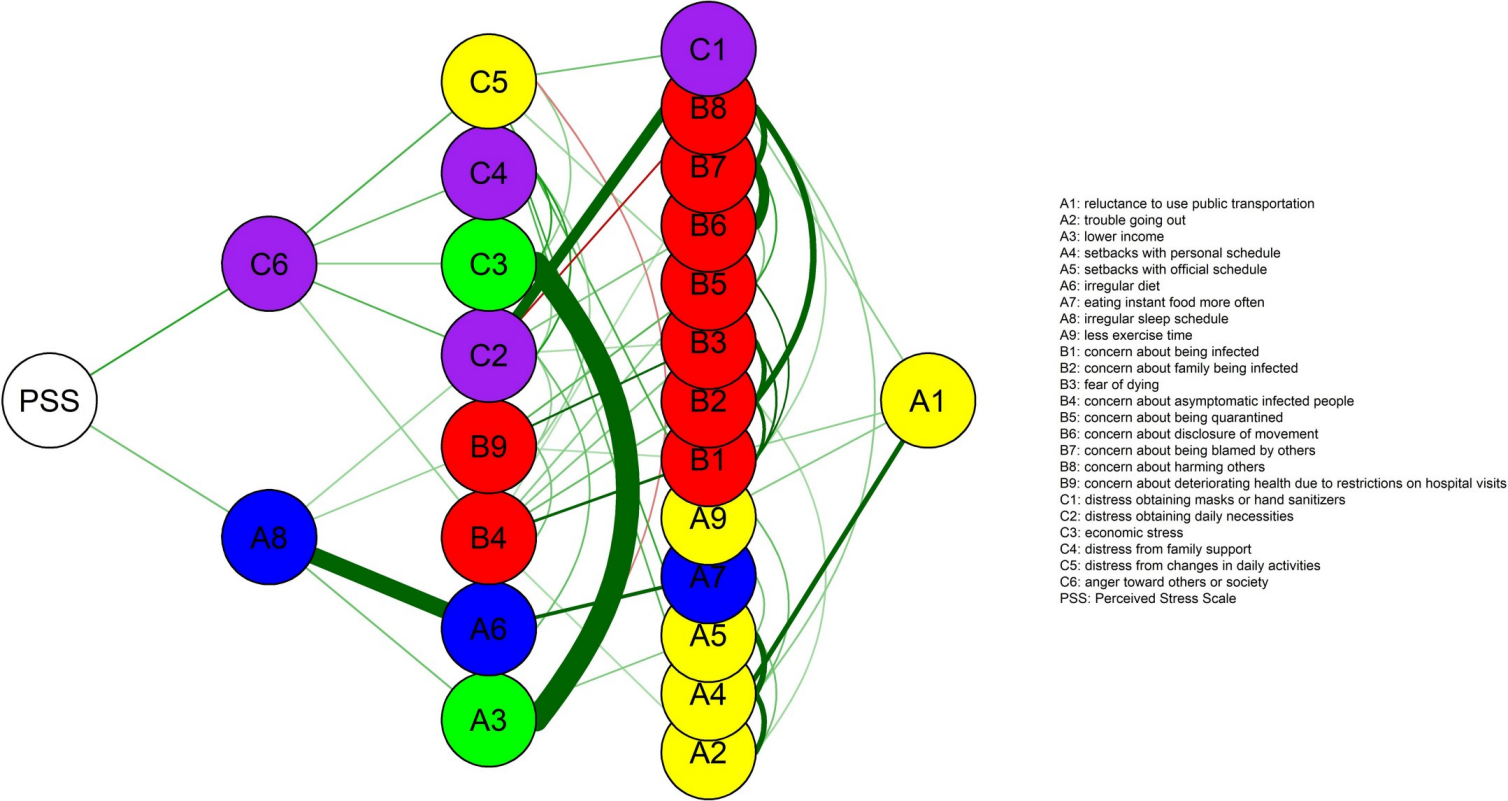
Flow diagram from the Perceived Stress Scale (PSS) node. A PSS node represents the total score of the 10-item PSS.

When the overall level of psychological stress (PSS node) was added to the network of responses to unusualness, this was primarily connected with ‘anger toward others or society’ (C6 node) and ‘irregular sleep schedule’ (A8 node). Subsequently, ‘anger toward others or society’ was connected to the ‘distress from changes in daily activities’ (C5 node), ‘distress from family support’ (C4 node), ‘economic stress’ (C3 node), ‘distress in obtaining daily necessaries’ (C2 node), and ‘concern about asymptomatic infected people’ (B4 node). ‘Irregular sleep time’ was also connected with ‘distress in obtaining daily necessities’ (C2 node), ‘concern about deteriorating health due to restrictions on hospital visits’ (B9 node), ‘irregular diet’ (A6 node), and ‘lower income’ (A3 node) nodes. A flow diagram from the PSS node is shown in Fig 3. The bootstrapped CI around the edge weight between PSS and C6 (PSS–C6) was smaller and did not contain zero, compared to that of the edge between PSS and A8 (PSS–A8). The CS coefficients were 0.672, 0.283, and 0.439, for node strength, closeness, and betweenness, respectively, reaching levels comparable to those of the network without the PSS node.

### 3.4. Comparison of networks between men and women

Overall, we did not observe much difference in the network structure of responses to unusualness between men and women (S4 Fig). The network invariance (Statistic = 0.203, P = 0.647) and global strength invariance (Statistic = 0.879, P = 0.258) were not statistically different between the sexes. We found no difference between sexes with regard to the centrality indices of most nodes, with the exception of the strengths of A7, B2, B3, and B9 nodes, the closeness of the B9 node, and the betweenness of the A5 node. The edge invariance test showed only a small number of edges, of which the strength differed between the sexes, including B6‒B8, B6‒C2, B3‒B9, and B5‒B6.

**Fig 4:**
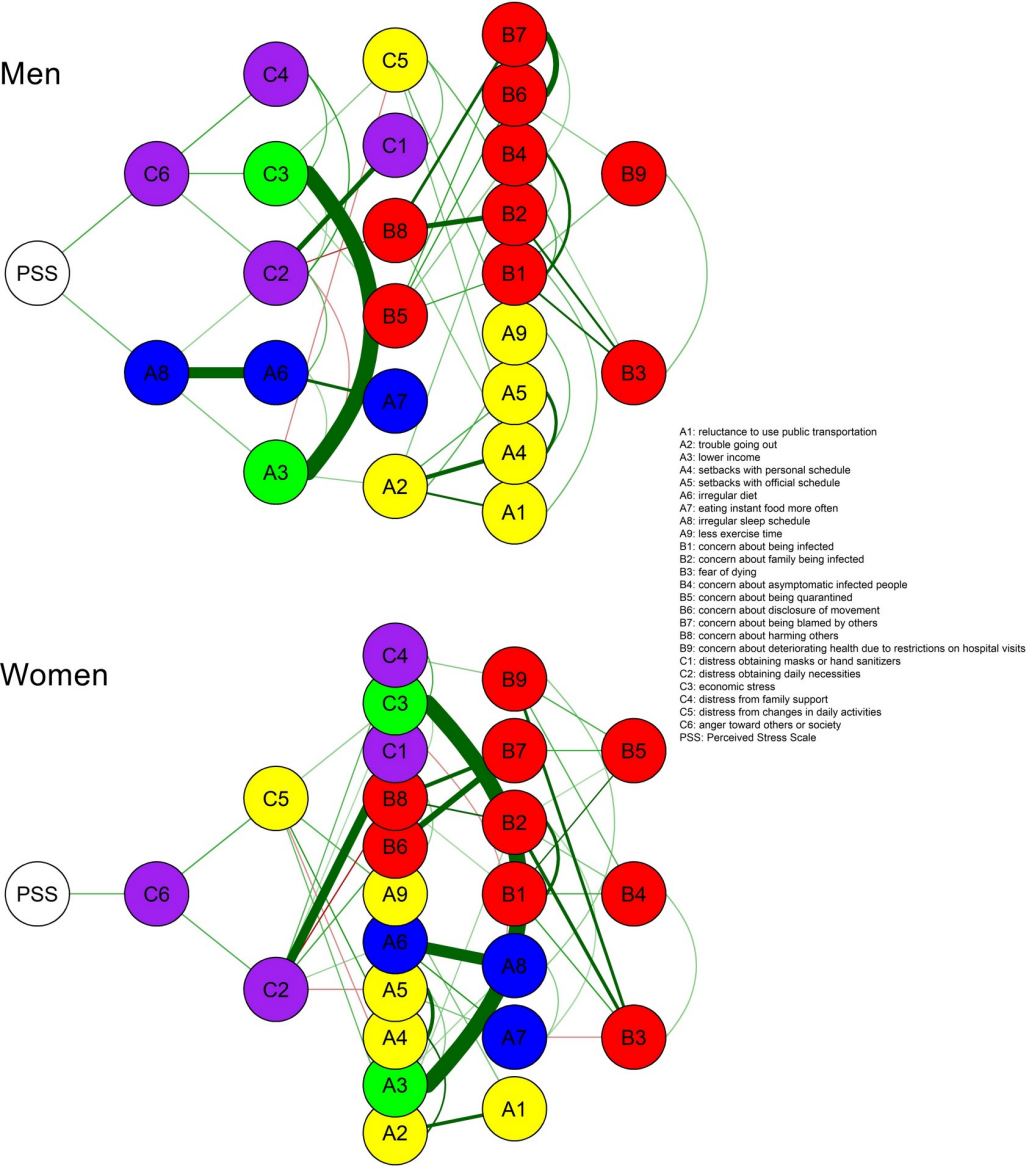
Flow diagrams from the Perceived Stress Scale (PSS) node for men and women. A PSS node represents the total score of the 10-item PSS.

When the overall level of psychological stress (PSS node) was added to the men and women’s networks of responses to unusualness, this was first connected with ‘anger toward others or society’ (C6 node) in both (Fig 4). Subsequently, ‘anger toward others or society’ was connected with ‘distress from family support’ (C4 node) and ‘economic stress’ (C3 node) in men only and ‘distress from changes in daily activities’ (C5 node) in women only. In addition, ‘anger toward others or society’ was connected with ‘distress in obtaining daily necessities’ (C2 node) in both men and women.

## 4. Discussion

We investigated the unusualness and the resulting distress experienced during the COVID-19 outbreak as a system of interconnected factors using network analysis. From a network perspective, human behaviors and outcomes can be conceptualized as emergent phenomena from a system of reciprocal interactions of relevant variables [38]. Network analysis is a promising method for examining the complex patterns of such relationships and generating graphical representations that conventional statistical methods cannot provide [13]. In recent years, network analysis has increasingly been applied in the fields of psychopathology, personality, and health psychology [40–43]. Accordingly, in this study, we expected that network analysis could offer novel insights into the behavior of individuals experiencing the COVID-19 pandemic.

The present study constructed a regularized partial correlation network of the unusualness and stress response experienced by Koreans during the COVID-19 outbreak and identified how their level of psychological stress was linked to the network. Centrality indices indicated that the ‘distress in obtaining daily necessities’ (C2 node) and ‘concern about harming others’ (B8 node) were the most centrally located nodes in the network, suggesting that these are the most important issues that are directly or indirectly correlated with many other issues encountered during the COVID-19 outbreak. When inputting the PSS-10 total score into the network, the overall level of psychological stress (PSS node) was primarily linked to ‘anger toward others or society’ (C6 node) and ‘irregular sleep schedule’ (A8 node), which were subsequently correlated mainly with nodes for ‘adaptive stress.’ In addition, psychological stress was indirectly connected to the nodes for ‘economic problems’ in men and ‘distress from changes in daily activities’ in women via the node for ‘anger toward others or society,’ suggesting that men and women may have different reasons for experiencing psychological stress during the COVID-19 outbreak.

### 4.1. Effects of insecurity regarding basic needs and collectivistic orientation on the psychosocial response to COVID-19

In terms of the global network metrics including network density and average clustering coefficient, 24 nodes did not appear to be tightly interconnected within the overall network structure. Instead, these nodes were grouped into five communities, namely, ‘fear of infection,’ ‘difficulty in outside activities,’ ‘economic loss,’ ‘disturbance in eating and sleeping,’ and ‘adaptive stress’ (Fig 1). In terms of centrality indices, the ‘distress in obtaining daily necessities’ (C2 node) and ‘concern about harming others’ (B8 node) were the most central nodes in the network of psychosocial responses to COVID-19 (Fig 2). In addition, the reliability of the network structure was confirmed by examining the accuracy of the edge weights and the stability of the centrality indices using the bootstrap method (S2 and S3 Figs).

The ‘distress in obtaining daily necessities’ (C2 node) was a hub node, which was highly connected with several nodes within the five communities with the highest values of node strength, closeness, and betweenness. As COVID-19 spread, people began to stockpile commodities such as food, bottled water, and toilet paper. Possible explanations for the phenomenon of panic buying during the COVID-19 outbreak include a sense of losing control over the environment and the future, self-preservation tendencies for the self and family, and individualism and competitiveness [44–47]. Distress from difficulties in obtaining daily necessities may be due to other people’s panic buying, but it also reflects feelings of insecurity and instability regarding basic needs, which could foster individual behavior such as stocking up on supplies. In this respect, feelings of insecurity related to basic needs can be considered one of the most influential issues among the psychosocial responses to COVID-19. Meanwhile, contrary to other countries, panic buying of daily necessities was not a significant issue in Korea [48]. Actually, in the current study, the majority of participants answered in the negative to the question of ‘distress in obtaining daily necessities,’ with a median score of 2 (disagree) (Table 1). Nevertheless, given that network analysis examines the entire pattern of connections between nodes, as opposed to a quantitative feature of an individual node, the finding of our network analysis indicates how much the issue of ‘distress in obtaining daily necessities’ was closely or highly correlated with other issues during the COVID-19 outbreak.

Estimated closeness and betweenness centrality indicated that the ‘concern about harming others’ (B8 node) was another central node in the network. This was closely linked to the ‘concern about family being infected’ (B2 node) and ‘concern about being blamed by others’ (B7 node) within the same community of ‘fear of infection.’ The ‘concern about harming others’ literally implies concern for others, and it contextually reflects collectivistic cultures in Korea. While anyone can become an asymptomatic carrier of the virus, many Koreans, who generally have family-centered and collectivistic orientation [49], seem to be worried about not only being infected themselves but also passing the virus to their family or others. This is supported by a recent report that Italian young adults showed higher concerns about their role as a possible asymptomatic carrier than being positive with COVID-19 themselves, suggesting their collectivistic orientations [50]. Among the nodes of other communities, the ‘concern about harming others’ (B8 node) was connected to the ‘setbacks with personal schedule’ (A4 node) and ‘distress in obtaining daily necessities’ (C2 node). Considering that the former was a centrally located node in the community of ‘difficulty in outside activities’ and the latter was a node with the highest centrality index in the whole network, connections with these nodes may contribute somewhat to the high closeness and betweenness of node B8 (‘concern about harming others’).

Interestingly, ‘distress in obtaining daily necessities’ (C2 node) and ‘concern about harming others’ (B8 node) were connected by a negative edge, suggesting that the two central nodes played contrasting roles in people’s responses to COVID-19 (Fig 1). Some evidence points to the potential role of individualism and collectivism in controlling the spread of COVID-19, suggesting that collectivism may play a more positive role in the adherence to behaviors aimed at reducing the spread of COVID-19 compared to individualism [50,51]. An individualistic orientation prioritizes individual needs above the group’s, whereas a collectivistic orientation focuses on group needs above the individual’s [52]. As mentioned above, ‘concern about harming others’ can be regarded as a typical collectivistic response. On the other hand, insofar as ‘distress in obtaining daily necessities’ reflects feelings of insecurity regarding basic needs and self-preservation tendencies, we can assume that this issue may be related to individualism. Thus, the negative connection between ‘distress in obtaining daily necessities’ and ‘concern about harming others’ may reflect the competing roles of insecurity about basic needs and a collectivistic orientation in the psychosocial response to COVID-19.

### 4.2. Role of unusualness due to COVID-19 on psychological stress and anger

When investigating how the overall level of psychological stress (PSS node) was associated with the network of response to unusualness under the COVID-19 outbreak, we found that this was linked directly to ‘anger toward others or society’ (C6 node) and ‘irregular sleep schedule’ (A8 node) (Fig 3). In particular, the bootstrapped CI indicated that the edge weight estimation between PSS‒C6 was more reliable than that of the PSS‒A8 edge. With regard to ‘anger toward others or society,’ people seemed to feel more frustration and express this more readily toward others or society than usual [53]. In fact, anger was one of the key emotions felt by people during COVID-19. According to a survey of 1,000 Koreans, which was conducted 2 months before the present study, public anger was growing as COVID-19 spread nationwide, and anger ranked second among the Korean public, following anxiety [54,55]. Increased frustration, irritability, and anger are common reactions to psychological stress, which in this case was exacerbated by the prevalent fear of infection and death, salary reductions and unemployment, restricted access to activities for coping with stress in healthy ways, and other unusual changes in daily life after the COVID-19 outbreak [56,57]. Following the PSS node, our findings also showed that ‘anger toward others or society’ (C6 node) was connected with several nodes: ‘difficulty in outside activities,’ ‘adaptive stress,’ ‘economic loss,’ and ‘fear of infection’ (Fig 3), suggesting that the prolonged restrictions on social activities and inconveniences of everyday life due to the outbreak response could play a more direct role in people’s anger than the fear of infection itself. In particular, we observed some differences in the type of nodes subsequently linked to the PSS‒C6 connection between men and women (Fig 4). Node C6, ‘anger toward others or society,’ was closely related to ‘distress from family support’ (C4 node) and ‘economic stress’ (C3 node) in men and ‘distress from changes in daily activities’ (C5 node) in women. Some recent studies have reported quantitative differences in psychological vulnerability to COVID-19 between men and women [58,59]. In contrast, our findings revealed qualitative sex differences in how daily life changes due to COVID-19 could affect psychological responses and mental health. Therefore, future research is needed to explore the psychological effects of COVID-19 with respect to sex.

### 4.3. Limitations

The present study had several limitations. First, our results do not represent the psychological responses of all Koreans to COVID-19, as the study participants lived in mostly in metropolitan areas with high population densities. Second, the survey tools used in this study were not fully validated due to the topic’s urgency. Third, because this network was established based on a cross-sectional survey conducted only 3 months after the spread of COVID-19 in Korea, the network structure may change over time, depending on the course of the pandemic. Fourth, considering the relatively low levels of the CS coefficients for node closeness and betweenness, these centrality indices should be interpreted with care. Fifth, the role of individualism and collectivism in the psychosocial response to COVID-19 offers a plausible explanation for the network models calculated here via exploratory network analyses. Moreover, other interpretations of our findings are also possible. Finally, the findings may be specific to Korea’s collectivist culture. Considering that cultural backgrounds may have a significant impact on the network of people’s responses to COVID-19, further network studies are needed to replicate our findings not only in other East Asian cultures, which, as in Korea, tend to be collectivistic, but also in Western cultures, which tend to be more individualistic.

### 4.4. Conclusion

To the best of our knowledge, this is the first network study to investigate the psychosocial response to the COVID-19 outbreak. Our results indicate that ‘distress in obtaining daily necessities’ and ‘concern about harming others’ were the most influential issues in the network of responses to the unusual situation due to COVID-19 in Koreans, suggesting that feelings of insecurity about basic needs and a collectivistic orientation play competing roles in their psychosocial responses. The psychological stress tended to invoke resentment and anger, particularly in relation to economic problems in men and restrictions in daily activities in women. Further network studies are needed to explore the adaptive responses to COVID-19 from different cultural backgrounds and from the perspective of mental health.

## Supporting information

S1 Fig Bootstrapped difference tests for centrality indices. Black boxes indicate nodes that differ significantly from other nodes. White boxes show the values for node strength, closeness, and betweenness.
(TIF)

S2 Fig Bootstrapped confidence intervals (CIs) around estimated edge weights of the network of the responses to unusualness due to COVID-19. The red line indicates the sample values, and the gray area corresponds to the bootstrapped CIs.
(TIF)

S3 Fig Stability of the centrality indices in the network of the responses to unusualness due to COVID-19. Lines indicate the average correlations between centrality indices of networks sampled with people dropped and the original sample. Areas indicate the 95% confidence interval.
(TIF)

S4 Fig Centrality indices of the network with respect to men and women’s responses to unusualness due to COVID-19. Centrality indices are shown as standardized z-scores.
(TIF)

S1 File (XLSX)

## References

[1] Covid-19 National Emergency Response Center, Epiemiology and Case Management Team, Korea Centers for Disease Control and Prevention. Coronavirus Disease-19: The First 7,755 Cases in the Republic of Korea. Osong Public Health Res Perspect. 2020; 11:85–90. 10.24171/j.phrp.2020.11.2.05 . 32257774 PMC7104685

[2] Ryu S, Chun BC, Korean Society of Epidemiology 2019-nCoV Task Force Team. An interim review of the epidemiological characteristics of 2019 novel coronavirus. Epidemiol Health. 2020; 42:e2020006. 10.4178/epih.e2020006 . 32023775 PMC7011107

[3] Haleem A, Javaid M, Vaishya R. Effects of COVID 19 pandemic in daily life. Curr Med Res Pract. 2020 10.1016/j.cmrp.2020.03.011 . 32292804 PMC7147210

[4] Emanuel EJ, Persad G, Upshur R, Thome B, Parker M, Glickman A, et al. Fair Allocation of Scarce Medical Resources in the Time of Covid-19. N Engl J Med. 2020; 382:2049–55. 10.1056/NEJMsb2005114 . 32202722

[5] Yoo JH, Hong ST. The Outbreak Cases with the Novel Coronavirus Suggest Upgraded Quarantine and Isolation in Korea. J Korean Med Sci. 2020; 35:e62. 10.3346/jkms.2020.35.e62 . 32030926 PMC7008072

[6] Nicola M, Alsafi Z, Sohrabi C, Kerwan A, Al-Jabir A, Iosifidis C, et al. The socio-economic implications of the coronavirus pandemic (COVID-19): A review. Int J Surg. 2020; 78:185–93. 10.1016/j.ijsu.2020.04.018 . 32305533 PMC7162753

[7] Jung SJ, Jun JY. Mental Health and Psychological Intervention Amid COVID-19 Outbreak: Perspectives from South Korea. Yonsei Med J. 2020; 61:271–2. 10.3349/ymj.2020.61.4.271 . 32233168 PMC7105405

[8] Kim S-W, Su K-P. Using psychoneuroimmunity against COVID-19. Brain, Behavior, and Immunity. 2020; 87:4–5. 10.1016/j.bbi.2020.03.025 32234338 PMC7194899

[9] Pfefferbaum B, North CS. Mental Health and the Covid-19 Pandemic. N Engl J Med. 2020 10.1056/NEJMp2008017 32283003

[10] Chew NWS, Lee GKH, Tan BYQ, Jing M, Goh Y, Ngiam NJH, et al. A multinational, multicentre study on the psychological outcomes and associated physical symptoms amongst healthcare workers during COVID-19 outbreak. Brain Behav Immun. 2020 10.1016/j.bbi.2020.04.049 . 32330593 PMC7172854

[11] Mazza C, Ricci E, Biondi S, Colasanti M, Ferracuti S, Napoli C, et al. A Nationwide Survey of Psychological Distress among Italian People during the COVID-19 Pandemic: Immediate Psychological Responses and Associated Factors. Int J Environ Res Public Health. 2020; 17 10.3390/ijerph17093165 . 32370116 PMC7246819

[12] Qiu J, Shen B, Zhao M, Wang Z, Xie B, Xu Y. A nationwide survey of psychological distress among Chinese people in the COVID-19 epidemic: implications and policy recommendations. Gen Psychiatr. 2020; 33:e100213. 10.1136/gpsych-2020-100213 . 32215365 PMC7061893

[13] Borgatti SP, Mehra A, Brass DJ, Labianca G. Network analysis in the social sciences. Science. 2009; 323:892–5. 10.1126/science.1165821 . 19213908

[14] Lavoie J, Douglas K. The Perceived Stress Scale: Evaluating Configural, Metric and Scalar Invariance across Mental Health Status and Gender. Journal of Psychopathology and Behavioral Assessment. 2012; 13:34–48. 10.1007/s10862-011-9266-1.

[15] Lee EH. Review of the psychometric evidence of the perceived stress scale. Asian Nurs Res (Korean Soc Nurs Sci). 2012; 6:121–7. 10.1016/j.anr.2012.08.004 . 25031113

[16] Cohen S. Perceived stress in a probability sample of the United States. The social psychology of health. The Claremont Symposium on Applied Social Psychology. Thousand Oaks, CA, US: Sage Publications, Inc; 1988. pp. 31–67.

[17] Lee EH, Chung BY, Suh CH, Jung JY. Korean versions of the Perceived Stress Scale (PSS-14, 10 and 4): psychometric evaluation in patients with chronic disease. Scand J Caring Sci. 2015; 29:183–92. 10.1111/scs.12131 . 24660854

[18] Epskamp S, Cramer AOJ, Waldorp LJ, Schmittmann VD, Borsboom D. qgraph: Network Visualizations of Relationships in Psychometric Data. Journal of Statistical Software. 2012; 48:1–18. 10.18637/jss.v048.i04 WOS:000305117400001.

[19] Chen JH, Chen ZH. Extended Bayesian information criteria for model selection with large model spaces. Biometrika. 2008; 95:759–71. 10.1093/biomet/asn034 WOS:000258861000017.

[20] Friedman J, Hastie T, Tibshirani R. Sparse inverse covariance estimation with the graphical lasso. Biostatistics. 2008; 9:432–41. 10.1093/biostatistics/kxm045 18079126 PMC3019769

[21] Foygel R, Drton M. Extended Bayesian information criteria for Gaussian graphical models. Advances in Neural Information Processing Systems, 23, 24th Annual Conference on Neural Information Processing Systems 2010, NIPS 20102010.

[22] Fruchterman TMJ, Reingold EM. Graph drawing by force-directed placement. Software: Practice and Experience. 1991; 21:1129–64. 10.1002/spe.4380211102.

[23] Golino H, Christensen A, Moulder R. EGAnet: Exploratory Graph Analysis–A framework for estimating the number of dimensions in multivariate data using network psychometrics. R package version 0.9.6 ed 2020.

[24] Pons P, Latapy M. Computing Communities in Large Networks Using Random Walks. J Graph Algorithms Appl. 2006; 10:191–218. 10.7155/jgaa.00124.

[25] Csardi G, Nepusz T. The Igraph Software Package for Complex Network Research. InterJournal. 2005; Complex Systems:1695.

[26] Borgatti SP, Cross R. A relational view of information seeking and learning in social networks. Management Science. 2003; 49:432–45. 10.1287/mnsc.49.4.432.14428 WOS:000182677200007.

[27] Mao GY, Zhang N. Analysis of Average Shortest-Path Length of Scale-Free Network. Journal of Applied Mathematics. 2013 WOS:000322241200001. 10.1155/2013/935154 24415902 PMC3886863

[28] Antoniou IE, Tsompa ET. Statistical analysis of weighted networks. Discrete Dynamics in Nature and Society. 2008 10.1155/2008/375452 WOS:000256785100001.

[29] Esfahlani FZ, Sayama H, Visser KF, Strauss GP. Sensitivity of the Positive and Negative Syndrome Scale (PANSS) in Detecting Treatment Effects via Network Analysis. Innov Clin Neurosci. 2017; 14:59–67. . 29410938 PMC5788252

[30] Borgatti SP. Centrality and network flow. Social Networks. 2005; 27:55–71. 10.1016/j.socnet.2004.11.008.

[31] Freeman LC. Centrality in social networks conceptual clarification. Social Networks. 1978; 1:215–39. 10.1016/0378-8733(78)90021-7.

[32] Kitsak M, Gallos LK, Havlin S, Liljeros F, Muchnik L, Stanley HE, et al. Identification of influential spreaders in complex networks. Nature Physics. 2010; 6:888–93. 10.1038/Nphys1746 WOS:000283715900022.

[33] Rodrigues F. Network Centrality: An Introduction. arXiv: Physics and Society. 2019:177–96.

[34] Opsahl T, Agneessens F, Skvoretz J. Node centrality in weighted networks: Generalizing degree and shortest paths. Social Networks. 2010; 32:245–51. 10.1016/j.socnet.2010.03.006.

[35] Bringmann L, Elmer T, Epskamp S, Krause R, Schoch D, Wichers M, et al. What Do Centrality Measures Measure in Psychological Networks? Journal of Abnormal Psychology. 2019; 128 10.1037/abn0000446 31318245

[36] Saramaki J, Kivela M, Onnela JP, Kaski K, Kertesz J. Generalizations of the clustering coefficient to weighted complex networks. Phys Rev E Stat Nonlin Soft Matter Phys. 2007; 75:027105. 10.1103/PhysRevE.75.027105 . 17358454

[37] Epskamp S, Borsboom D, Fried EI. Estimating psychological networks and their accuracy: A tutorial paper. Behav Res Methods. 2018; 50:195–212. 10.3758/s13428-017-0862-1 . 28342071 PMC5809547

[38] Hevey D. Network analysis: a brief overview and tutorial. Health Psychology and Behavioral Medicine. 2018; 6:301–28. 10.1080/21642850.2018.1521283 WOS:000472538400018. PMC8114409 34040834

[39] van Borkulo C, Boschloo L, Kossakowski J, Tio P, Schoevers R, Borsboom D, et al. Comparing network structures on three aspects: A permutation test. 2017. www.researchgate.net/publication/314750838. Cited 10 August 2020. 10.1037/met0000476 35404628

[40] Borsboom D. A network theory of mental disorders. World Psychiatry. 2017; 16:5–13. 10.1002/wps.20375 . 28127906 PMC5269502

[41] Kossakowski JJ, Epskamp S, Kieffer JM, van Borkulo CD, Rhemtulla M, Borsboom D. The application of a network approach to Health-Related Quality of Life (HRQoL): introducing a new method for assessing HRQoL in healthy adults and cancer patients. Qual Life Res. 2016; 25:781–92. 10.1007/s11136-015-1127-z . 26370099 PMC4830856

[42] Murphy J, McBride O, Fried E, Shevlin M. Distress, Impairment and the Extended Psychosis Phenotype: A Network Analysis of Psychotic Experiences in an US General Population Sample. Schizophr Bull. 2018; 44:768–77. 10.1093/schbul/sbx134 . 29036519 PMC6007708

[43] Richetin J, Preti E, Costantini G, De Panfilis C. The centrality of affective instability and identity in Borderline Personality Disorder: Evidence from network analysis. PLoS One. 2017; 12:e0186695. 10.1371/journal.pone.0186695 . 29040324 PMC5645155

[44] Arafat SMY, Kar SK, Marthoenis M, Sharma P, Hoque Apu E, Kabir R. Psychological underpinning of panic buying during pandemic (COVID-19). Psychiatry Res. 2020; 289:113061. 10.1016/j.psychres.2020.113061 . 32413711 PMC7202808

[45] Bavel JJV, Baicker K, Boggio PS, Capraro V, Cichocka A, Cikara M, et al. Using social and behavioural science to support COVID-19 pandemic response. Nat Hum Behav. 2020; 4:460–71. 10.1038/s41562-020-0884-z . 32355299

[46] Garbe L, Rau R, Toppe T. Influence of perceived threat of Covid-19 and HEXACO personality traits on toilet paper stockpiling. PLoS One. 2020; 15:e0234232. 10.1371/journal.pone.0234232 . 32530911 PMC7292383

[47] Sim K, Chua HC, Vieta E, Fernandez G. The anatomy of panic buying related to the current COVID-19 pandemic. Psychiatry Res. 2020; 288:113015. 10.1016/j.psychres.2020.113015 . 32315887 PMC7158779

[48] Lee J. Why is there no panic buying in Korea?: KOREA.NET; 2020. Available from: http://www.korea.net/NewsFocus/Society/view?articleId=184370.

[49] Cha J-H. Aspects of individualism and collectivism in Korea. Individualism and collectivism: Theory, method, and applications. Cross-cultural research and methodology series, Vol. 18. Thousand Oaks, CA, US: Sage Publications, Inc; 1994. pp. 157–74.

[50] Germani A, Buratta L, Delvecchio E, Mazzeschi C. Emerging Adults and COVID-19: The Role of Individualism-Collectivism on Perceived Risks and Psychological Maladjustment. Int J Environ Res Public Health. 2020; 17 10.3390/ijerph17103497 . 32429536 PMC7277425

[51] Xiang P, Zhang H, Geng L, Zhou K, Wu Y. Individualist-Collectivist Differences in Climate Change Inaction: The Role of Perceived Intractability. Front Psychol. 2019; 10:187. 10.3389/fpsyg.2019.00187 . 30809166 PMC6379328

[52] Hofstede G, Hofstede GJ, Minkov M. Cultures and Organizations: Software of the Mind, Third Edition: McGraw-Hill Education; 2010.

[53] Lwin MO, Lu J, Sheldenkar A, Schulz PJ, Shin W, Gupta R, et al. Global Sentiments Surrounding the COVID-19 Pandemic on Twitter: Analysis of Twitter Trends. JMIR Public Health Surveill. 2020; 6:e19447. 10.2196/19447 . 32412418 PMC7247466

[54] Park J-Y. More S. Koreans feel angry at coronavirus news as situation prolongs: Hankyoreh; 2020. Available from: http://english.hani.co.kr/arti/english_edition/e_national/931284.html.

[55] You M-S. Second survey of the South Korean public’s risk perceptions: Hankook Research; 2020. Available from: https://hrcopinion.co.kr/archives/15221.

[56] Park SC, Park YC. Mental Health Care Measures in Response to the 2019 Novel Coronavirus Outbreak in Korea. Psychiatry Investig. 2020; 17:85–6. 10.30773/pi.2020.0058 . 32093458 PMC7047003

[57] Torales J, O’Higgins M, Castaldelli-Maia JM, Ventriglio A. The outbreak of COVID-19 coronavirus and its impact on global mental health. Int J Soc Psychiatry. 2020; 66:317–20. 10.1177/0020764020915212 . 32233719

[58] Rodriguez-Rey R, Garrido-Hernansaiz H, Collado S. Psychological Impact and Associated Factors During the Initial Stage of the Coronavirus (COVID-19) Pandemic Among the General Population in Spain. Front Psychol. 2020; 11:1540. 10.3389/fpsyg.2020.01540 . 32655463 PMC7325630

[59] Varshney M, Parel JT, Raizada N, Sarin SK. Initial psychological impact of COVID-19 and its correlates in Indian Community: An online (FEEL-COVID) survey. PLoS One. 2020; 15:e0233874. 10.1371/journal.pone.0233874 . 32470088 PMC7259495

