# Supplementary figures and images for: Network study of responses to unusualness and psychological stress during the COVID-19 outbreak in Korea
Source: PLoS One. 2021 Feb 26;16(2):e0246894. doi: 10.1371/journal.pone.0246894 (PMC7909677; doi:10.1371/journal.pone.0246894)

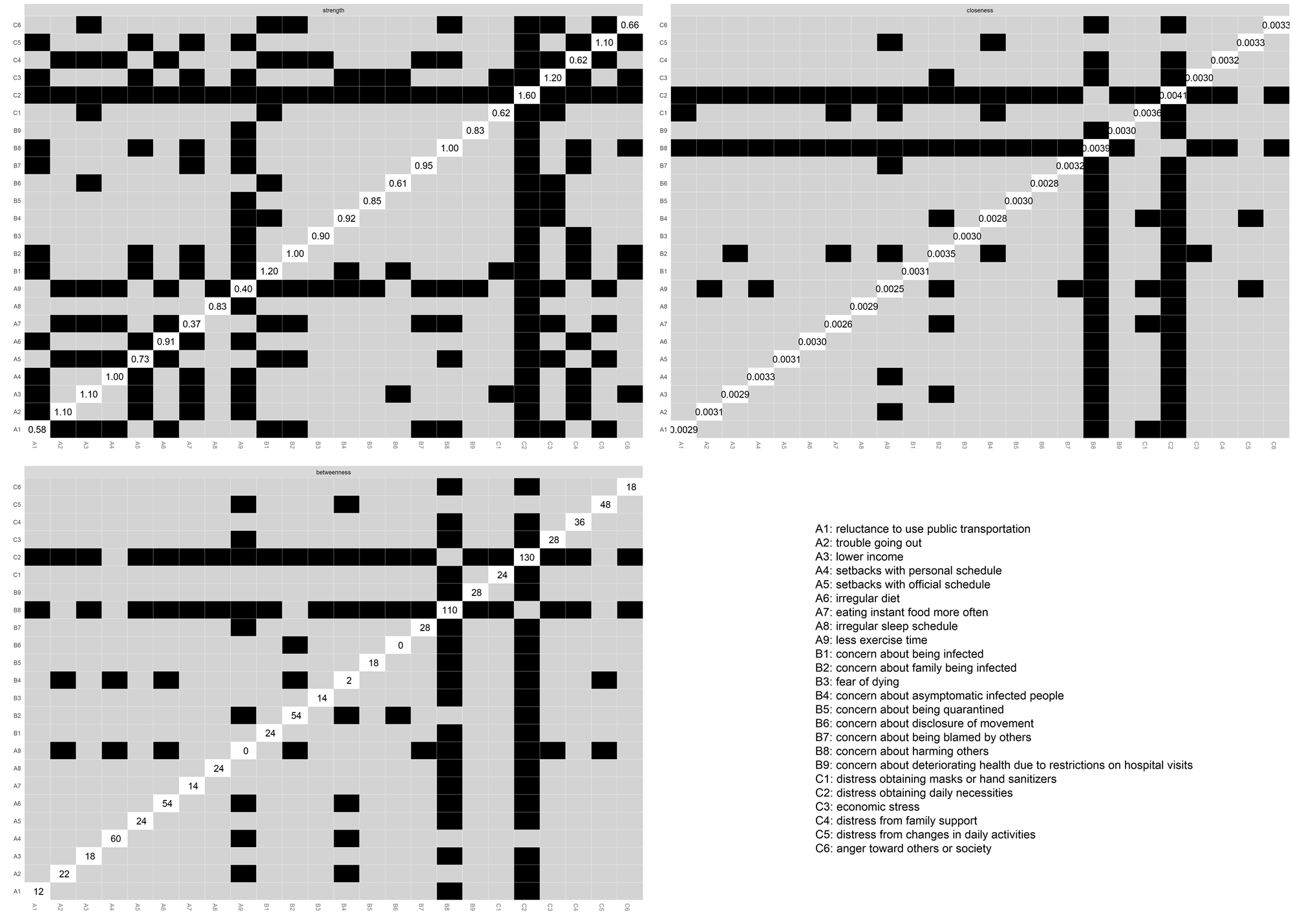

Supplement: S1 Fig — Black boxes indicate nodes that differ significantly from other nodes. White boxes show the values for node strength, closeness, and betweenness. (TIF) [file pone.0246894.s001.tif]

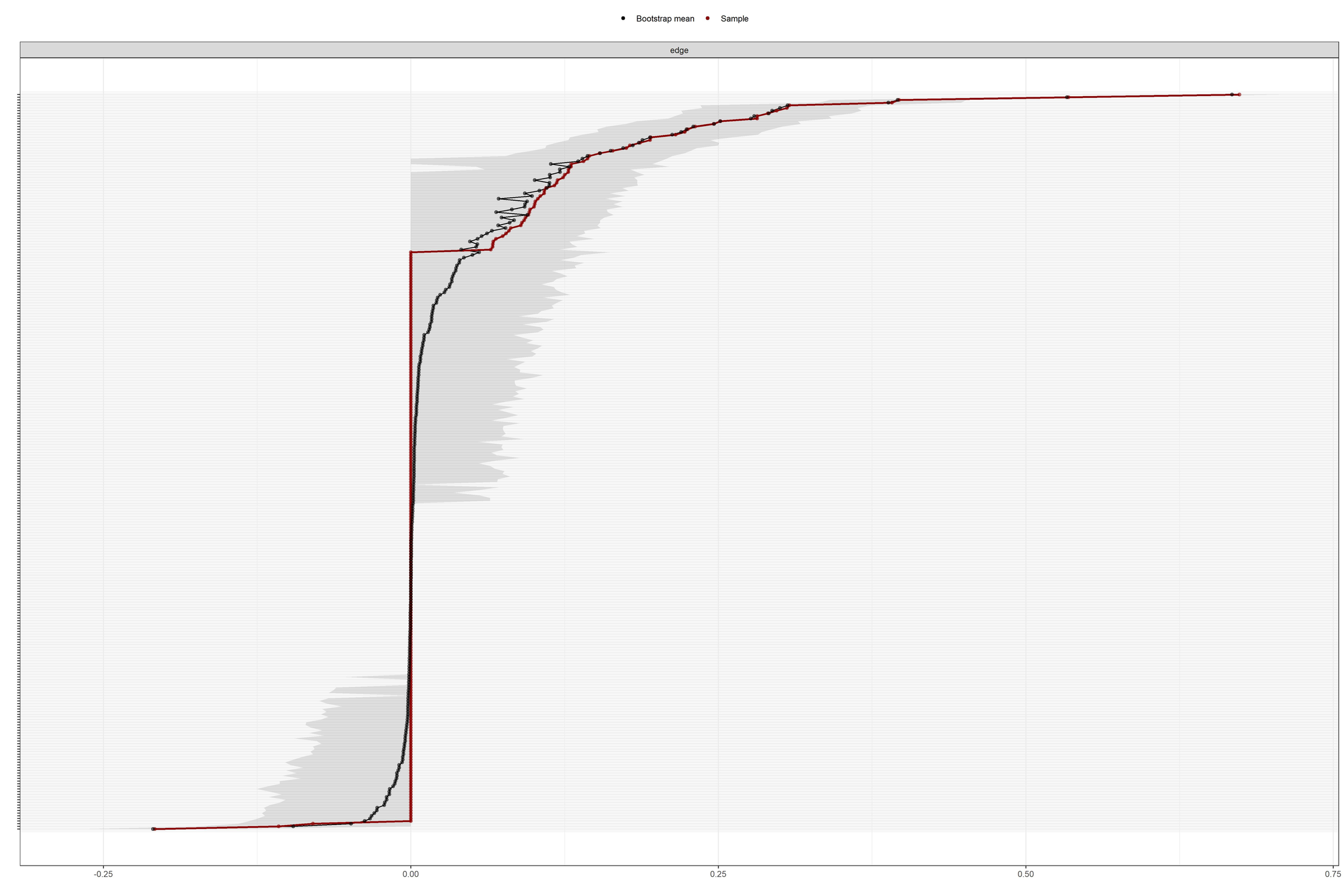

Supplement: S2 Fig — The red line indicates the sample values, and the gray area corresponds to the bootstrapped CIs. (TIF) [file pone.0246894.s002.tif]

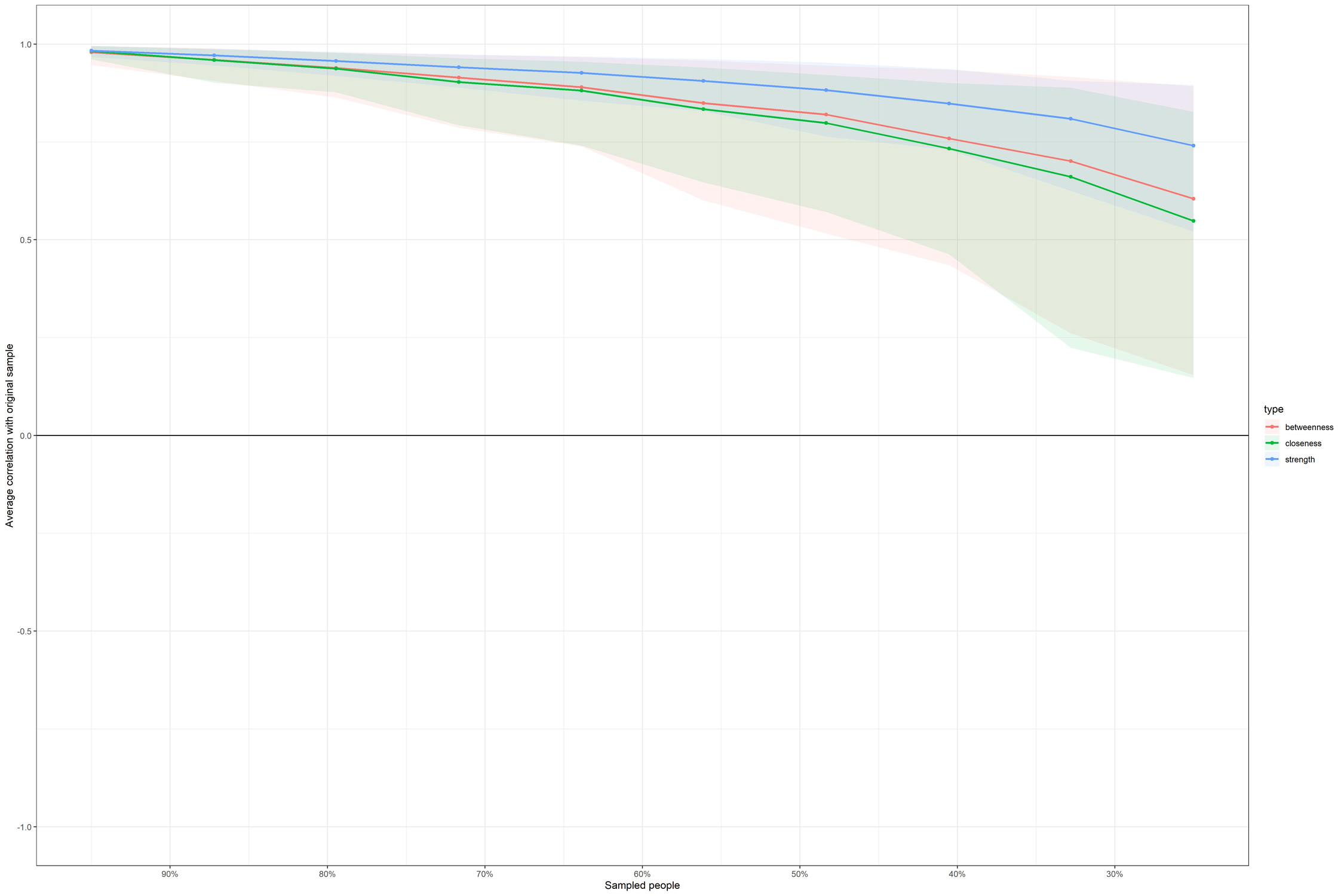

Supplement: S3 Fig — Lines indicate the average correlations between centrality indices of networks sampled with people dropped and the original sample. Areas indicate the 95% confidence interval. (TIF) [file pone.0246894.s003.tif]

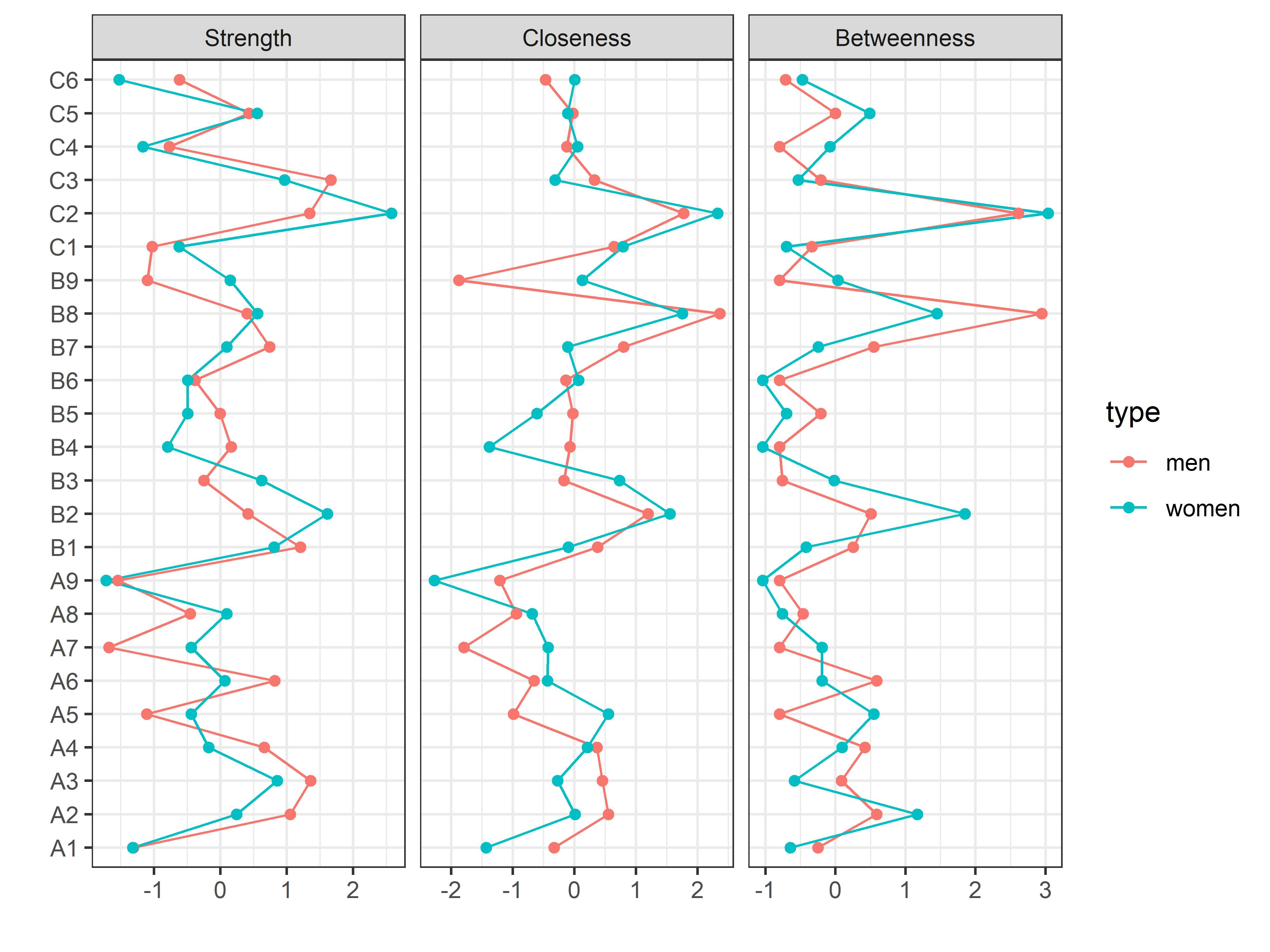

Supplement: S4 Fig — Centrality indices are shown as standardized z-scores. (TIF) [file pone.0246894.s004.tif]
